# A Study of the Printability of Alginate-Based Bioinks by 3D Bioprinting for Articular Cartilage Tissue Engineering

**DOI:** 10.3390/polym14020354

**Published:** 2022-01-17

**Authors:** Izar Gorroñogoitia, Uzuri Urtaza, Ana Zubiarrain-Laserna, Ana Alonso-Varona, Ane Miren Zaldua

**Affiliations:** 1 Leartiker S. Coop., 48270 Makina-Xemein, Spain; igorronogoitia@leartiker.com (I.G.); uurtaza@leartiker.com (U.U.); anzubiarrain@leartiker.com (A.Z.-L.); 2 Faculty of Medicine and Dentistry, University of the Basque Country (UPV/EHU), 48940 Leioa, Spain; ana.alonsovarona@ehu.eus

**Keywords:** alginate, hydrogel, bioprinting, printability, scaffold

## Abstract

Three-dimensional bioprinting combined with natural hydrogels is a promising technology for the treatment of several pathologies and different tissue regeneration. One of the most studied tissues is cartilage, a complex and avascular tissue that displays a limited self-repair capacity after injuries. Herein, the development of alginate-based hydrogels and scaffolds containing different microstructure is presented and the printability of alginate by 3D bioprinting is studied. Rheological characterization was performed for the determination of viscosity and viscoelastic properties of hydrogels and mechanical characterization was carried out for the determination of compressive modulus of alginate hydrogels. All these characteristics were correlated with alginate behaviour during 3D bioprinting process. For the printability evaluation filament diameter, perimeter of the pores, area of the pores and shrinkage of alginate scaffolds were measured. The results demonstrate that alginate microstructure has a great influence on its printability and on hydrogels’ physicochemical properties. Molecular weight of alginate determines its viscosity while M/G ratio determines cross-linking conditions and mechanical properties that vary with cross-linking density. These results suggest the importance of an exhaustive control of the viscoelastic and mechanical properties of alginate hydrogels to obtain structures with high resolution and precision.

## 1. Introduction

Nowadays due to the many injuries of articular cartilage (AC), its reparation has a big demand and is a big challenge since it does not have the ability to repair itself, even if several surgical and nonsurgical techniques do exist to repair it. AC injuries are quite difficult to be fully cured and need time. AC is a viscoelastic tissue where its main functions are to cushion the overload of surfaces in contact and to allow the displacement of bone surfaces throughout movement. During its regeneration, a fibrocartilage tissue is formed which presents different mechanical and biological properties from that of native tissue [1]. Therefore, tissue engineering is an alternative and attractive approach for the treatment of cartilage since it tries to mimic the functional and biological features of cartilage native tissue. It consists of selecting suitable cells, biomaterials and designing suitable scaffolds. These materials should match both appropriate mechanical and biological properties for a successful performance of the cartilage regeneration. There is a vast number of biomaterials that can be used for the regeneration of cartilage such as alginate, gelatin, collagen, hyaluronic acid, etc. All of them have similar structure to the extracellular matrix (ECM) and act as supportive 3D structures for cell growth. One of the most studied hydrogels has been alginate, which comprises appropriate features for cartilage tissue application [1,2,3]. It is a natural hydrophilic polymer derived from brown algae that is composed of β-D-mannuronic acid (M) and α-L guluronic acid (G) monomers via covalent bonds. The chemical composition of alginates depends on its origin since the molecular weight (M_w_), configuration (distribution along the polymeric chain) and proportion of these two monomers vary significantly according to algae species and harvesting place [4,5,6,7,8,9]. The monomer M is responsible of providing flexibility while G monomer provides rigidity to the structure [1,3,6,8]. Consequently, it is a critical factor to study the composition of alginate in detail, to control its mechanical and biological properties. One of the abilities of alginate is the formation of a hydrogel by ionic crosslinking. A divalent cation (Ca^2+^) reacts with the carboxylic (COO^−^) groups of G-blocks of alginates forming a hydrogel that presents high-water absorbance capacity, soft nature and porous structure [1,5,6,8,9,10], thus allowing the diffusion of nutrients and oxygen, which are essential for the proliferation of cells. In addition, alginate possess degradability and biocompatibility as well as shear thinning behaviour, versatility and tunability, making it ideal for 3D bioprinting applications [2,3,5]. Farokhi et al. [1] highlighted the potential of alginate as the main component for the fabrication of different kinds of scaffolds (hydrogel, sponge, fibre, 3D printed) for articular cartilage tissue engineering. Rastogi and Kandasubramanian [2] reported the process of 3D extrusion bioprinting of alginate hydrogels considering its pre-/during/post-processing parameters. There are many other reports in which more than one type of alginate containing different chemical compositions (M/G ratio, M_w_) are studied in order to investigate the influence of alginate microstructure in the rheological and mechanical properties [6,7,8,10,11,12,13]. All of them agree that the elastic modulus (mechanical properties) is determined mainly by the M/G ratio of alginates and to a minor extent by the gelling ion (Ca^2+^) concentration. They report that concentration and M_w_ of alginate play a significant role in the flow behaviour and stiffness of alginates as well. However, all these reports do not study the influence of alginate molecular structure in the printability of them, when they are extruded by a 3D bioprinter.

There are already several reports of alginates in bioprinting [14,15,16,17,18,19,20,21]. All authors demonstrated the use of alginate as bioink for 3D scaffold fabrication by means of extrusion printing. The most important parameters that determine the printability of alginates are, on one hand, bioprinter-related parameters such as diameter and velocity of nozzle, air pressure, distance and temperature of platform and syringe. On the other hand, alginate-related parameters such as composition of alginate (Mw, M/G), concentration and gelling agent play an important role in its printability, thus making them key factors for a successful and high-resolution structure fabrication. Naghieh et al. [22] evaluated the printability of alginate-based hydrogels, in which they defined a series of parameters (strand printability (P_f_), pore printability (P_p_), pore irregularity) that determined the quality of printed structures, and Habib et al. [23] used a specific geometry to quantify the printability of constructs.

It should be noted that there are some contradictions about the relation of alginate chemical composition and its mechanical properties. Even though the large majority of the reports agree on the fact that alginates with high G content present higher stiffness and thus, higher modulus than low G content alginates [7,8,10,13], there are some studies that go against these statements. Suarez-Arnedo et al. [6] addressed that high-G alginates presented higher Young modulus (E’) but lower storage modulus (G’) than low-G alginates, when the elastic behaviour (G’) of stiff structures should be higher. It is true, though, that they do not consider the M_w_ of alginate samples, which also has an influence in the mechanical properties. Yeung and Kennedy [11] reached the conclusion that both high-G and low-G alginates have similar mechanical properties, but again, they do not consider the M_w_ of samples either. and different viscosity values that the different alginate samples present can be observed. According to Enobakhare et al. [8], the pore and network size of high-G alginate gels are bigger, thus enhancing the diffusion of molecules. However, high-G alginates should present smaller pore and network size because the cross-linking density should be higher (more cross-links in the network).

Due to the inconsistencies observed in the bibliography, it was decided to carry out this study that will be focused on the effect that the intrinsic properties of alginate (M_w_, M/G) have on its printability during extrusion through the needle. The printability is directed by the physicochemical properties, so the exhaustive control of the viscoelastic and mechanical properties of alginate is vital to obtain structures with high resolution and precision. The M_w_ and concentration will determine the viscosity of alginates and thus, the ability of the hydrogel to flow (flow-behaviour) during printing as well as the stability (rigidity) of the structure after printing. The M/G ratio will affect gelling conditions and mechanical properties of scaffolds that will vary according to cross-linking density, the latter also being affected by the gelling agent (Ca^2+^) concentration.

Rheological and mechanical properties of different type and concentration of alginates will be reported in this study with the aim of selecting and identifying the most suitable alginate to use as a bioink for 3D printing application. For that purpose, four sodium alginates with different microstructures were selected.

## 2. Materials and Methods

Sodium alginate low viscosity (W201502 and 180947), sodium alginate high viscosity (A2033 and 71238) and calcium chloride (CaCl_2_, anhydrous) were purchased from Sigma-Aldrich, St. Louis, MO, USA. The supplier does not provide data about the M_w_ and M/G ratio of alginates; therefore, they were characterized by POLYMAT (Donostia-San Sebastián, Spain). Table 1 shows the corresponding data (MWD, M/G) of each alginate. M/G ratio was determined by ^1^H-NMR in solution-state at high temperature (90 °C) in a Bruker AVANCE 500 spectrometer. Prior to analysis, samples were subjected to a mild partial acid hydrolysis to a diverse pH value with the aim of reducing the molecular weight of samples and enabling a proper characterization. This process was carried out according to the procedure reported by Salomonsen et al. [24]. Hydrolysed alginate samples were dissolved in D_2_O. The content of G and M was calculated based on the position of distinct signals in the anomeric region and their relative areas, which contain specific information about the chain composition. Molecular weight and polydispersity were determined by gel permeation chromatography (GPC) with a pump, automatic injector, a precolumn (Ultrahydrogel guard column, Waters, Milford, MA, USA) together with 3 columns in series (Ultrahydrogel 2000, Ultrahydrogel 250, Ultrahydrogel 120, Waters, Milford, MA, USA) and a refractive index detector (Optilab T-Rex, Wyatt Technology, Santa Barbara, CA, USA). The mobile phase for the measurements was 0.1 M NaNO_3_ at a flow-rate of 0.6 mL/min, and tests were performed at 35 °C. Alginate samples were dissolved in the mobile phase at a concentration of 2 mg/mL and were injected in the columns. Data were analysed using ASTRA 6 software (Wyatt Technology). Distribution of molecular weight (MWD) was obtained from a calibration curve that was performed by injecting various polyethylene glycol (PEO) standards in the range of 1970–318,000 Da. A three-letter acronym was designated to describe the sample with low G content, LoG, intermediate G content, InG and high G content, HiG. The subscripts refer to the M_w_ (in kDa) of the alginate.

### 2.1. Hydrogel Preparation and Crosslinking Method

Sodium alginate solutions were prepared with distilled water with concentrations ranging from 2 to 8% (*w*/*w*). In the case of high M_w_ sodium alginates, solutions were prepared up to 5 and 7% (*w*/*w*) of concentration due to the very high viscous solutions. Polymer powders were vigorously mixed under a propeller-type stirrer at room temperature until complete dissolution. The solutions were stored at 2–8 °C to prevent polymer degradation. Alginate bulk gels were prepared by immersing the samples in a 100 mM CaCl_2_ bath for 24 h. Gels were also cross-linked with 50 and 150 mM of CaCl_2_, but the most suitable was 100 mM. After the complete gelling of samples, the ones for compression testing were punched using a hole puncher with a diameter of 5 mm and the ones for viscoelastic measurements with a diameter of 20 mm.

### 2.2. Rheological Characterization

Shear-viscosity of all hydrogel precursors was measured as a function of shear-rate (flow-curve experiments) at different sodium alginate concentrations and different temperatures (23, 30, 37 °C) using a HAAKE MARS III rheometer with parallel plates (20 mm, aluminium, gap = 1 mm). Shear-rate varied from 0.5 to 1000 s^−1^. Three measurements were performed with each concentration and temperature to evaluate statistical significance.

The flow behaviour index (n) data values were processed from the rheogram of each sodium alginate solution (not shown) and using the Ostwald–de Waele model [11] shown in Equation (1), where *τ* is the shear-stress (Pa), $\dot{\gamma }$ is the shear-rate (s^−1^) and parameters *K* and *n* are flow consistency index and flow behaviour index.

(1) $$\tau =K{\dot{\gamma }}^{n}$$

**Figure 1:**
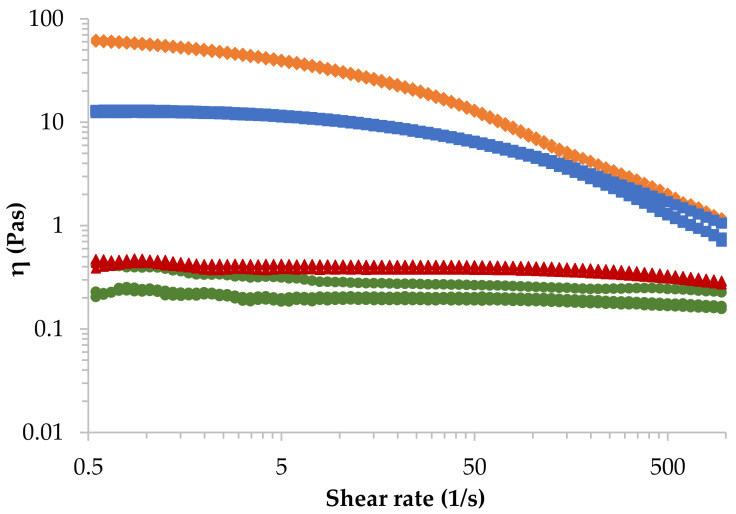
Shear-viscosity of four sodium alginate aqueous solutions at a concentration of 4% (*w*/*w*) and 23 °C; LoG_495_ (rhomb), HiG_427_ (square), InG_253_ (triangle), and LoG_187_ (sphere) (*n* = 3 per group).

Zero-shear viscosity (*η*_0_) data was obtained and analysed from the experimental flow-curves shown in Figure 1 and was calculated using the Carreau–Yasuda model [25] (Equation (2)), where *η* is the shear-viscosity, *η_∞_* is the infinite-shear viscosity, *τ* the relaxation time of the fluid, $\dot{\gamma }$ the shear-rate, *n* the flow index and *a* is a constitutive parameter.

(2) $$\eta =\eta_{\infty }+\frac{\left(\eta_{0}-\eta_{\infty }\right)}{{\left[1+{\left(\tau \dot{\gamma }\right)}^{a}\right]}^{\frac{1-n}{a}}}$$

Dynamic shear tests (time sweep experiments) were performed for the determination of storage modulus (G’) of each alginate gel at different concentrations using parallel plates (20 mm, aluminium serrated, F_n_ 2 and 5 N) at 23 °C. Time varied from 0 to 300 s applying a frequency of 1 Hz and a constant deformation in the linear viscoelastic regime (0.01%). All measurements were performed three times to facilitate statistical analysis.

### 2.3. Mechanical Characterization

The mechanical properties of alginate gels presenting different M_w_ and M/G ratio were determined by applying unconfined compression (UC) at different alginate concentrations using a universal testing machine (Bionix MTS Insight^TM^) equipped with a 50 N load cell (MTS Systems). For UC testing (*n* = 5 per group), a pre-load of 0.01 N was first applied and then ramp compression tests to 75% of strain at a rate of 1 mm/min in air at room temperature were carried out. Tangent modulus at a target strain of 10% was determined for each hydrogel.

### 2.4. Fabrication Process of the Scaffold

Scaffolds of 0.84 × 30 × 30 mm dimensions and 5 mm of pore size were printed by means of a BIO V1 3D Bioprinter (REGEMAT 3D, Granada, Spain) equipped with three syringes and one FDM extruder, consisting of hardware and Designer software (REGEMAT 3D, Granada, Spain) that are connected by an electronic control unit (ECU). Scaffolds were fabricated layer by layer (4 layers) in a glass bed that was at room temperature (RT) using a 5 mL syringe, 27 G conical nozzle inner diameter, 5 mm/s of nozzle speed, 0.5 mm of offset and the flow-rate was set to 1 µL/s for all alginate solutions except for LoG_187_ that was set to 1.5 µL/s. After printing, scaffolds were immersed in a 100 mM CaCl_2_ solution for 24 h. To check the uniformity of the fabricated scaffolds, 3 imprints were made with each alginate solution.

### 2.5. Printability Evaluation

The printability of hydrogels was addressed in a geometry characterization assessment with the aim of selecting the best hydrogel candidate for biofabrication based on material properties. Therefore, 3D studies were performed to check the quality and printability of the scaffolds (4 layers). This characterization was performed in two different ways; first, the filament width, pore area and the perimeter of the pores were measured using NIS-elements (Nikon Instruments Inc. 3.2002.738.1500, Melville, NY, USA) software with a magnification of 0.61× before and after immersing the scaffolds in a 100 mM CaCl_2_ solution for 24 h. The aforementioned parameters were determined according to the procedure reported by Naghieh et al. [22]; primarily, the standard strand diameter (*D_s_*) was calculated, which was equivalent to strand width and was compared with the experimental strand diameter (*D_exp_*). These calculations were performed using the following equations:

(3) $$\rho =\frac{Mass}{Volume}$$

(4) $$Q=\frac{Volume}{Time}$$

(5) $$Nozzle\;speed=\frac{4Q}{\pi {\left(D_{s}\right)}^{2}}$$

where *ρ*, *Q* and *D_s_* are density, flow rate and standard strand diameter. Therefore, alginate solutions were dispensed in a Petri Dish using BIO V1 3D Bioprinter (REGEMAT 3D, Granada, Spain) from a syringe (5 mL) with 27 G needle for a certain period (20 s) and a flow-rate of 1 µL/s and nozzle speed was set to 5 mm/s. The dispensed material was weighted using an analytical balance (COBOS^®^, HR-250AZ). From Equation (4), the volume is calculated and later the flow rate (*Q*) and strand diameter (*D_s_*) can be calculated for a specific flow-rate and nozzle speed. Once calculating *D_s_*, strand printability was defined as:

(6) $$Strand\;printability,\;(P_{f})=1-\frac{D_{s}-D_{exp.}}{D_{s}}$$

Pore printability was evaluated with the following equation:

(7) $$Pore\;printability,\;(P_{p})=\frac{p^{2}}{16\beta }$$

where *β* and *p* are the area and perimeter of a pore of a scaffold.

After this evaluation, with the aim of a more representative and real study, experimental strand diameter was compared among the different hydrogel precursors exhibiting different properties and pore size was compared with the theoretical values considering the dimensions of the scaffolds fabricated (5 × 5 mm of pore size). Finally, the shrinkage of the hydrogels during post-crosslinking was also considered in the evaluation for the printability. The latter was calculated using the following equation [26]:

(8) $$\bar{S}=\frac{{\bar{S}}_{x}+{\bar{S}}_{y}}{2}=\frac{\left(\frac{{\bar{L}}_{bx}\;-\;{\bar{L}}_{ax}}{{\bar{L}}_{bx}}\;*100\;\%\right)+\left(\frac{{\bar{L}}_{by}\;-\;{\bar{L}}_{ay}}{{\bar{L}}_{by}}*100\;\%\right)}{2}\;$$

where ${\bar{S}}_{x}$ and $\;{\bar{S}}_{y}$ are the arithmetic means of the shrinkage in x-direction and y-direction, ${\bar{L}}_{bx}$ and ${\bar{L}}_{by}$ are the averaged side length in the respective direction before cross-linking and ${\bar{L}}_{ax}$ and ${\bar{L}}_{ay}$ after cross-linking.

## 3. Results

### 3.1. Characterization of the Different Sodium Alginates

Compositional properties, such as molecular weight and the proportion of M and G monomers, are essential characteristics for the determination of the alginate behaviour during bioprinting process. Molecular weight influences the viscosity of alginate solution; therefore, it will determine the ability of alginate to flow during the extrusion process. If viscosity is too low, there will be dripping of the fluid and a non-constant fluid will be obtained, leading to non-uniform structures and high probability of scaffold collapse. At the same time, if viscosity is too high clogging of the tip can happen and cell viability could be compromised. According to Ouyang et al. [27], cells die from 800 Pa of shear stress. Hence, viscosity should be high enough to attain constant fluid and uniform and stable structures, but low enough to avoid clogging of the tip and enable cell viability.

Regarding M/G ratio of alginate, it will affect the cross-linking conditions and thus, mechanical properties of alginate gels since they will be determined by the cross-linking density. The higher the G content, the higher the degree of cross-linking, therefore, enhanced mechanical properties and stable structures with high-shape fidelity after printing will be obtained, avoiding scaffold collapse.

#### 3.1.1. Rheological Characterization

Figure 1 represents the shear viscosity of four alginate solutions presenting different molecular weight at 4% (*w*/*w*) and 23 °C obtained from the rotational shear-test experiments. It can be observed that alginates presenting high molecular weight (rhomb and square symbols) exhibit a pseudoplastic (shear-thinning) behaviour, viscosity decreases as the shear rate increases. This behaviour is more pronounced when molecular weight of alginate increases, and it is a fundamental characteristic for 3D extrusion bioprinting. A reduction in viscosity at high shear rates is preferred to avoid cells to die during extrusion. However, in the case of low molecular weight alginates (triangle and dash symbols), Newtonian behaviour is observed in almost the whole shear rate range, obtaining a very slight shear-thinning behaviour at very high shear rates. Consequently, problems of collapse are foreseen for those alginates. The effect of the molecular weight is very clear, and the solution of LoG_495_ alginate (rhomb) shows the highest shear-viscosity since it presents the highest molecular weight.

In Table 2, the Ostwald–de Waele parameters obtained from the power-law model (Equation (1)) and Newtonian viscosity obtained from Carreau–Yasuda model (Equation (2)) of all sodium alginates in aqueous solution at 4% (*w*/*w*) at three different temperatures are presented. As it can be observed, the value of *n* decreases with molecular weight, which means that the LoG_495_ alginate solution will present greater molecular disentanglements when subjected to shear forces. All the samples, though, display shear-thinning behaviour (*n* < 1) confirming the results in Figure 1. In the case of LoG_187_ and InG_253_ *n* is very close to 1, indicating that their behaviour is almost Newtonian. According to the zero-shear viscosity (*η_0_*), it increases with molecular weight exhibiting greater deviations at higher molecular weights. All these values agree with the fact that when sodium alginate is dissolved in water, on one hand, polymer chains have less freedom to move in the solution when the chain length (molecular weight) is longer because they have less space, thus, the viscosity increases. On the other hand, the polymeric chains form physical entanglements, which are temporary and depend on the molecular weight. When the molecular weight increases, the probability of forming entanglements is higher since the chains are longer and join another chain when they are moving, therefore, viscosity increases as well as the deviations in measurements.

Regarding temperature effect, in Table 2, it can be observed that it has very little effect on viscosity. This effect increases with molecular weight of alginate, where temperature energy would break the physical entanglements formed in the solution, but the effect is quite insignificant. Therefore, the temperature at which the bioprinting process will be performed will not affect the printability of the hydrogel precursors.

#### 3.1.2. Viscoelastic and Mechanical Characterization

**Figure 2:**
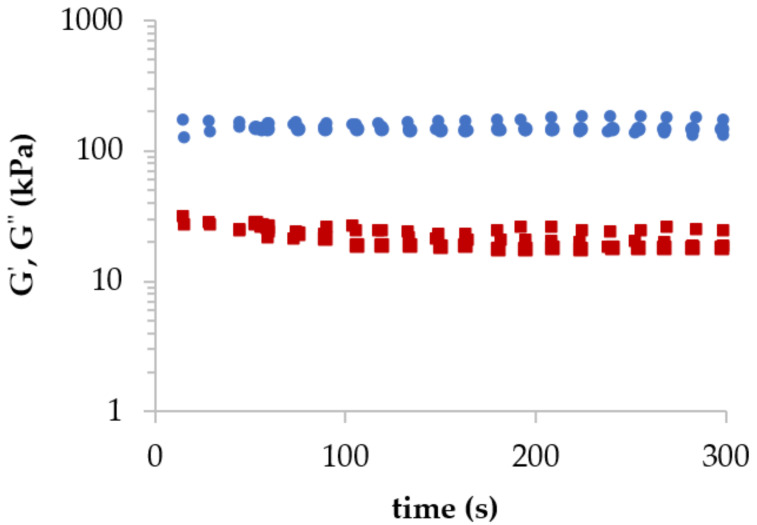
Storage modulus G’ (circles) and loss modulus G’’ (squares) as a function of time for HiG_427_ alginate gel at 4% (*w*/*w*) of alginate concentration and 100 mM of CaCl_2_ at 23 °C (*n* = 3).

In Figure 2, the dynamic behaviour of 4% (*w*/*w*) HiG_427_ alginate after complete gelation with CaCl_2_ is shown. The behaviour of the hydrogel is fully elastic with storage moduli G’ (circle) independent of the applied time (constant across the whole time-range). In addition, storage modulus G’ (circle) was higher than the loss modulus G’’ (square) across the whole time-interval.

**Figure 3:**
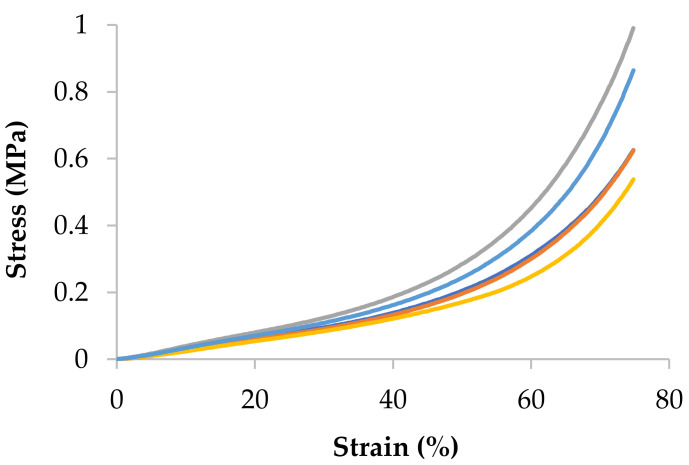
Stress–strain curves for HiG_427_ alginate gel at 4% (*w*/*w*) and 100 mM of CaCl_2_ at 23 °C (*n* = 5).

**Figure 4:**
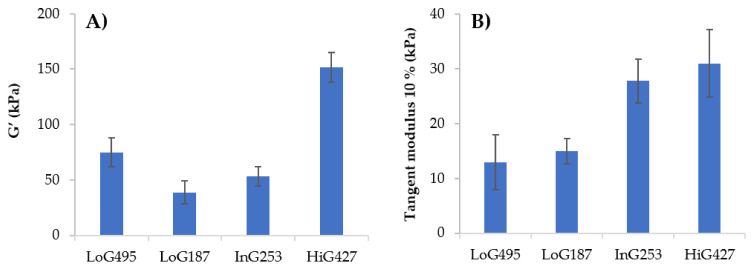
Values of the (**A**) storage modulus G’ and (**B**) tangent modulus at 10% of strain for each alginate gel at 4% (*w*/*w*) of alginate concentration and 100 mM of CaCl_2_ at 23 °C.

**Figure 5:**
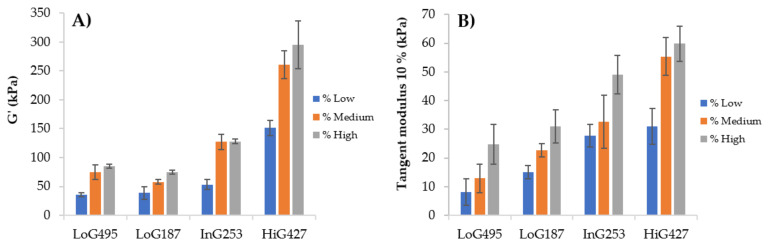
Values of the (**A**) storage modulus G’ and (**B**) tangent modulus at 10% of strain for each alginate gel at different alginate concentrations and 100 mM of CaCl_2_ at 23 °C.

Alginate gels’ compression modulus was determined from the stress–strain curves, shown in Figure 3 that were obtained from unconfined compression test experiments. It can be observed the non-linear behaviour of alginate gels, in fact it presents rather a rubbery behaviour. For this reason, the Young modulus cannot be calculated so tangent modulus will be considered, specifically, tangent modulus at a target strain of 10%. As an example, the HiG_427_ alginate is presented in Figure 3, but the rest of the data are collected in Figure 4B and Figure 5B.

It is already known in literature that the ionic cross-linking of sodium alginate takes place between the guluronate (G) blocks of alginate and the Ca^2+^ divalent ion forming a well-known structure called an egg-box. Therefore, for a constant CaCl_2_ concentration, the higher the G content of a sodium alginate, the higher the crosslinking density of the formed gel, resulting in a stiffer gel. Therefore, mechanical properties of the gels will be determined by the cross-linking density of the alginate, while at the same time, the latter will be determined by the G% of alginate.

In Figure 4, the storage modulus (G’) and tangent modulus at 10% of strain for each sodium alginate gel at 4% (*w*/*w*) of concentration and 100 mM of CaCl_2_ at 23 °C are shown. Generally, as G content increases in alginate chemical composition, higher cross-linking density and thus, higher storage modulus of the gel is obtained. Tangent modulus increases with alginate G content as well, as shown in Figure 4B. Briefly, cross-linking density of alginate is influenced by the M/G ratio, but M_w_ should also play a role in the mechanical properties of the gel. For instance, in this work an exception occurs with LoG_495_, in which the length of the chains is so long that it will allow the generation of physical entanglements in addition to the chemical ones (ionic bonds), thus contributing to the viscoelastic behaviour of the hydrogel and obtaining higher storage modulus than LoG_187_ (Figure 4A), even if it has lower G. Now, if LoG_495_ and HiG_427_ alginates are compared, they both exhibit similar M_w_. Nevertheless, because of the great difference in G%, HiG_427_ alginate presents higher storage and tangent modulus, confirming that the effect of M/G ratio in mechanical properties is much greater than that of the M_w_. At the same time, LoG_187_ and InG_253_ show similar M_w_, but the latter shows higher storage and tangent modulus because of the greater G% in the chemical composition. These results agree with the statements reported in literature about the cross-linking mechanism of sodium alginate, in which they highlight the relation of G content with viscoelastic and mechanical properties of alginate gels [7,10,13].

#### 3.1.3. Optimization of the Final Hydrogel

For the optimization of the final hydrogel, the influence of alginate concentration on physicochemical properties was considered. This could help to modulate the properties of the hydrogel, leading to an optimization of the final ink for 3D bioprinting application.

In Table 3, the Ostwald–de Waele parameter values and zero-shear viscosity of four alginate-based hydrogel precursors at different concentrations and at 23 °C are shown. It can be observed that the value of *K* and *η*_0_ increases with concentration as well as deviations in measurements. On the contrary, *n* values decrease with concentration, obtaining higher pseudoplastic behaviour at higher concentrations and therefore, more molecular disentanglements. When concentration is higher, the number of polymeric chains in the solution increases dramatically, leading to a reduced mobility of the chains since the space is reduced and at the same time, it is more likely for the chains to form physical entanglements because they are closer from each other. Consequently, viscosity increases with concentration. It is true, though, that the increase in viscosity is negligible for low M_w_ alginate, LoG_187_, where the viscosity increases only from 0.3 to 1.4 Pas when the concentration is doubled. The opposite occurs with high M_w_ alginates, in the case of LoG_495_, when the concentration is doubled the viscosity is 30 times higher. Working with high M_w_ samples (HiG_427_ and LoG_495_) turned out to be much more difficult to handle because they took more time to dissolve in water, obtaining very viscous solutions, therefore, it was not possible to prepare solutions of 8% (*w*/*w*) with these samples.

Figure 5 represents the values of the storage modulus (G’) and tangent modulus at 10% of strain for each alginate gel at different alginate concentrations at 23 °C using a concentration of 100 mM of CaCl_2_. It can be observed that concentration of alginate does have a great influence in the storage modulus (G’) and tangent modulus due to a greater viscosity at higher concentrations that causes an increase in the gels’ moduli.

### 3.2. Printability Evaluation

Printability evaluation was carried out by selecting certain alginate solutions that enabled a comparison between the different hydrogel precursors. Therefore, all different sodium alginates at 4% (*w*/*w*) of concentration were chosen for the printability evaluation. Apart from these ones, the 6% (*w*/*w*) of HiG_427_ alginate was also selected for the printability evaluation since it presented good rheological and mechanical properties for 3D bioprinting. All these alginate solutions showed viscosities in the range of 0.3–100 Pas, which was the appropriate range for a good processability according to He et al. [28]. The remaining solutions were considered too concentrated that they would cause a major shear-stress for cells during the extrusion process, and at the same time, they would cause a diffusion barrier of nutrients and lack of cell migration due to a higher solid content in the solution.

**Figure 6:**
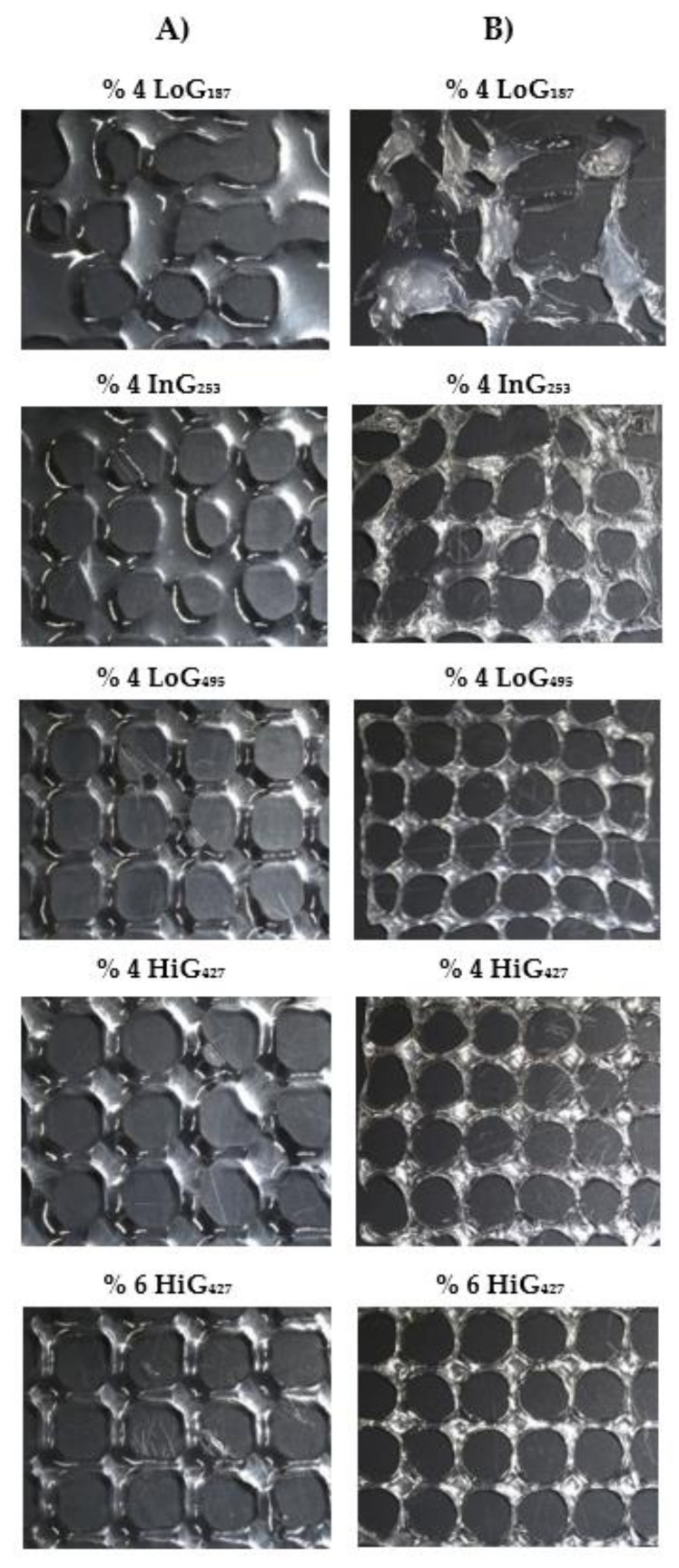
Different alginate imprint structures: (**A**) uncross-linked samples, (**B**) cross-linked samples (Magnification: 0.61×, Needle inner diameter: 27 G).

Figure 6A shows the different uncross-linked imprint structures using different alginates at 4 and 6% (*w*/*w*) of concentration and a needle inner diameter of 27 G. By simple visual examination it could be clearly observed that LoG_187_ alginate was not printable since no scaffold was obtained due to the low viscosity of the hydrogel precursor that was not able to maintain any shape fidelity and consequently, collapsed. InG_253_ alginate, which exhibited slightly higher viscosity than LoG_187_, presented better printability, but still some filaments of the structure collapsed after printing, forming a very wide strand diameter and irregularities in the pores. With respect to LoG_495_ alginate, due to its high viscosity, nice structures were obtained with very uniform square-like pores and well-defined filament across the whole imprint structure. Finally, HiG_427_ alginate showed good printability too, obtaining uniform and regular pores. A difference could be noticed between the 4% and 6% alginate samples mainly in the shape of the pores in which round-like pores were formed with 4% hydrogel precursor that was attributed to the collapse of the vertices of the pores due to its lower viscosity and slightly higher pooling. A 6% alginate sample, however, displayed square-like pores, indicating higher viscosity, and thus better ability to maintain the shape. It should be noted that the vertices of the structures are very marked due to a higher material accumulation on those points since the nozzle goes twice through each vertex to make each layer of pore, obtaining a greater height than by design. LoG_495_ presenting a viscosity of 76 Pas and 6% HiG_427_ alginate exhibiting a viscosity of 60 Pas at 23 °C, displayed best imprint structures, obtaining square-like uniform pores with good shape-fidelity in both cases.

The same imprint structures as in Figure 6A but cross-linked with 100 mM of CaCl_2_ during 24 h are shown in Figure 6B. Generally, a simple visual inspection reveals that all alginate samples suffered from a notable shrinkage since more pores of the structure were visible in the images using the same magnification (0.61×). At the same time, after cross-linking the pores took a round-like shape. As mentioned before, with LoG_187_ and InG_253_ alginate samples, no stable scaffold was obtained, confirming their lack of printability. LoG_495_ was easier to handle, but because of its very low G content, it was quite soft and tended to bend, decreasing its shape-fidelity. Moreover, it suffered the highest shrinkage, concluding that cross-linking of alginate, in particular the ionic bonds between calcium and guluronic blocks of alginate, helps preserve the original shape and size of the imprint structure. Otherwise, pores were quite uniform. Finally, HiG_427_ was the easiest one to handle due to its high viscosity and high G content in the chemical composition that enable to obtain structures with good enough rigidity and consistency. In this case, uniform pores were obtained, especially for the 6% hydrogel precursor.

Table 4 shows printability parameters for uncross-linked (not shady) and cross-linked (shady) imprint structures with three replicates with each alginate group. As mentioned before, LoG_187_ and InG_253_ were not printable due to their very low viscosity, so no printability parameters were measured. It can be observed that strand printability (P_f_) and pore printability (P_p_) were very similar for all uncross-linked hydrogel precursors, which indicates how unrepresentative these parameters are in order to evaluate the printability of the different hydrogels exhibiting different properties. In this case, P_f_ (Equation (6)) is calculated following the procedure reported by Naghieh et al. [22], where they calculate from theoretical strand diameter (*D_s_*), which the latter is calculated considering solely the density of the alginate solution as the intrinsic property of the material and does not consider the viscosity, die swelling nor the surface tension of the print bed (glass bed), which are critical parameters to define the strand diameter of a hydrogel precursor [29,30,31]. For the same reason, the P_f_ values obtained were far from 1 (the closer to 1 the better printability), indicating that alginate solutions suffered from post-extrusion swelling and partially wetted the print bed once they were printed. Finally, it must be said that these samples are not crosslinked with CaCl_2_ causing bigger collapse of the strand. According to P_p_ (Equation (7)), also calculated as Naghieh et al. [22], it does not consider the collapse of the vertices that form the pores, which is the cause of obtaining smaller or bigger pores that can vary with alginate viscosity. With respect to crosslinked structures, again, P_f_ and P_p_ were not representative to evaluate the printability for the same reasons as before, and apart from that, because *D_s_* was calculated without considering the shrinkage of the structure due to the cross-linking of alginate, which makes it an even more unrepresentative evaluation method.

Therefore, a comparison of the experimental strand diameters (*D_exp_*) was made between the different uncross-linked hydrogel precursors, and the perimeter (p) and area (β) of the pores were compared with the intended dimensions of the scaffolds fabricated. In the printing process during the extrusion of the filament, two opposite phenomena are responsible for the experimental filament diameter, *D_exp_*: (1) Die-swelling is bigger at high M_w_ resulting in a wider *D_exp_* and (2) at higher M_w_ the viscosity is higher, so the collapse of the filament is smaller, resulting in a narrower *D_exp_*. In the case of LoG_495_, the alginate solution exhibited highest M_w_, thus, highest viscosity; however, its *D_exp_* was higher than HiG_427_ possibly due to a higher die swelling that was caused due to its high molecular weight. HiG_427_ alginate solution presented narrower *D_exp_* confirming that the viscosity of this solution is high enough not to collapse too much. Slight difference could be seen between the 4% (viscosity = 13 Pas at 23 °C) and 6% (viscosity = 60 Pas at 23 °C) alginate samples where slightly narrower *D_exp_* was obtained for the 6% hydrogel precursor, which displayed higher viscosity and thus, was able to maintain the shape better.

With respect to scaffolds cross-linked after printing, as mentioned before, lower values for printability parameters were obtained since they suffered from a big shrinkage. For example, before cross-linking LoG_495_ alginate hydrogel showed a pore area of 14.10 mm^2^ and after cross-linking dropped to half its value (6.97 mm^2^). Printability of cross-linked alginate hydrogels was evaluated, making a comparison between them and considering the theoretical dimensions of the scaffolds and the shrinkage, calculated as Hazur et al. [26]. LoG_495_ alginate hydrogel presented highest shrinkage (36%), therefore, the smallest strand diameter, perimeter and pore area were obtained, moving far from theoretical values. HiG_427_ alginate hydrogel had the best printability, especially the 6% alginate hydrogel, which showed the highest pore size and strand diameter, obtaining a minor shrinkage (17%) than 4% alginate hydrogel (24%), possibly due to its higher viscosity (58.9 Pas instead of 12.9 Pas) that helped to maintain the shape-fidelity of the imprint structure.

## 4. Discussion and Conclusions

Alginate is one of the most used biopolymers for bioprinting since it is biodegradable, biocompatible and does not induce inflammatory response [32]. However, the data reported in literature so far has not highlighted the influence that alginate chemical composition (M_w_ and M/G ratio) can have in the extrusion printing process. Most of the time, it is blended with other biomaterials [18,23,33,34,35,36,37], therefore, they do not consider its structure as a crucial intrinsic property for bioprinting. This paper aims to demonstrate the importance that alginate microstructure has on its physicochemical properties since they govern not only the behaviour of alginate during printing and post-printing, but also the biological response of the final hydrogel. Therefore, herein we characterized four sodium alginates presenting different molecular structures; two of them exhibited low molecular weight with low G content and intermediate G content, and the remaining two displayed high molecular weight with high G content and low G content. This allowed us to develop hydrogel precursors and scaffolds with different rheological and mechanical properties. In extrusion bioprinting, viscosity of the hydrogel precursor will determine the formation of a continuous filament or droplet and at the same time the structural stability of the printed filament once deposited. In Table 3, can be noticed that the viscosity is determined by the molecular weight. With the results obtained, LoG_187_ and InG_253_ alginate solutions exhibited a non-constant flow during extrusion and drops were formed because they present very low M_w_, making them unsuitable for 3D bioprinting. Furthermore, their viscosity could not be modulated with concentration. However, LoG_495_ and HiG_427_ alginate solutions presented constant flow and uniform filaments were formed since they present M_w_ high enough to ensure stability and uniformity. In addition, they were characterized by the shear-thinning behaviour, avoiding cell damage during extrusion and thus, making them suitable for this application. Apart from this, in the case of high M_w_ alginates, a little change in concentration leads to a high change in viscosity, so this helps to adjust the final properties. Another important point is that from batch to batch the M_w_ and G/M ratio of alginate samples vary because they are natural polymers, so properties should be adjusted depending on the batch to obtain target properties.

Regarding mechanical properties, based on the results obtained in the mechanical and viscoelastic assays, in Figure 4 it could be confirmed that mechanical properties are determined by the M/G ratio of alginate. In this context, although LoG_495_ was appropriate for bioprinting in terms of viscosity, due to the low G content, poorer mechanical properties were obtained with this alginate sample, therefore, slightly unstable structures were obtained. HiG_427_ alginate was the most suitable one for 3D bioprinting since it presented the highest tangent modulus and highest storage modulus, therefore, it was able to maintain the shape of the structures after printing and in addition, it has a molecular weight high enough to have an appropriate viscosity during 3D printing process. The effect of the concentration should also be considered since it can help to adjust the final properties of alginate gels. The higher the alginate concentration, the higher the modulus was.

With respect to bioprinting assays, the results are in accordance with rheological and mechanical characterization results obtained previously. LoG_187_ alginate presenting very low viscosity and G content was not printable at all because no scaffold could be obtained. InG_253_ alginate hydrogel exhibiting low viscosity and intermediate G content had very poor printability, making obtaining soft scaffolds very difficult to handle and with lot of irregularities in the pores. LoG_495_ had mediocre printability with uniform pores in the imprint structures, but the G content was so low that the scaffolds were soft and bent easily. Moreover, the shrinkage was so high that it negatively affected its printability, which caused more structural fineness and ease of breaking. Finally, the HiG_427_ alginate showed the best printability due to its high M_w_ and G content in the chemical composition where stiff scaffolds with uniform pores were obtained. It could also be concluded that the viscosity range (0.3–100 Pas) proposed by He et al. [28] was too wide for a good processability since poor or no printability was obtained with alginates presenting low viscosity (LoG_187_ = 0.3 Pas and InG_253_ = 0.4 Pas), which is in accordance to Park et al. [38], who mentioned that bioinks with < 10 Pas of zero-shear viscosity could not form stable structures. Therefore, with the aim of adjusting the suitable viscosity range for a good printability at these specific conditions, it would be 10–80 Pas. It is true though that with the alginate sample presenting a viscosity of 13 Pas at 23 °C, good imprint structures were obtained, but way better scaffolds were fabricated with alginates presenting viscosity range from 50–80 Pas. In addition, high G content in alginate structure would favour printability of fabricated structures because less shrinkage would be obtained.

Among the developed hydrogels, HiG_427_ alginate was the best candidate for bioprinting, as shown in the results. In spite of its appropriate M_w_ and G content that allowed us to develop hydrogels with good rheological and mechanical properties, we did not reach the modulus of the native articular cartilage (0.38 MPa) [39]. In fact, our values are quite far, showing a value of 0.06 MPa for the stiffest alginate hydrogel. With the aim of achieving hydrogels with better mechanical properties, double-network hydrogels have great potential looking to the future, but they need to be optimized to meet the needs of biological manufacturing. It consists of combining two biomaterials and cross-linking them to form interpenetrating (IPN) networks [40,41,42] with enhanced mechanical properties.

With the alginate being a renewable resource material, the authors’ main goal in this work was to control the influence of its microstructure in the processability, that is to analyse the relationship between microstructure of alginates, the rheological and viscoelastic properties, and identify their influence on printability. However, another important aspect to consider is the cellular characterization of the developed hydrogels. As these alginates are intended to be the basis for bioinks, further studies on cell survival during bioprinting, as well as on cell viability and proliferation, are necessary. The alginate with high viscosity that shows good printability will probably have lower cell viability. In contrast, low viscosity alginate may have poor printability but high cell viability, so a balance between both characteristics will be needed. Therefore, HiG_427_ alginate is the best one for 3D bioprinting applications, but it should be analysed in terms of biocompatibility and cell viability. Although this field is not the object of study of this work, it is very important to design and fabricate scaffolds with good biological properties to obtain successful regeneration of articular cartilage. Thus, further biological studies will be necessary to validate these hydrogels as possible candidates for the articular cartilage regeneration.

## Figures and Tables

**Table 1:** Data obtained from GPC (PEO standards) and ^1^H-NMR analysis for each alginate.

Sodium Alginate	Algae Species	Designation	GPC			^1^H-NMR		
			M_n_ (kDa)	M_w_ (kDa)	MWD	M (%)	G (%)	M/G
A2033	Macrocystis pyrifera	LoG_495_	249	495	2.0	65.8	34.2	1.9
W201502	Macrocystis pyrifera	LoG_187_	67	187	2.8	59.4	40.6	1.5
180947	Unknown	InG_253_	91	253	2.8	49.2	50.8	1.0
71238	Laminaria Hyperborea	HiG_427_	186	427	2.3	39.6	60.4	0.7

**Table 2:** Ostwald–de Waele parameters and zero-shear viscosity for all sodium alginates in aqueous solution at 4% (*w*/*w*) and three different temperatures.

Sodium Alginate	T (°C)	*K* (Pas)	*n*	*η*_0_ (Pas)
LoG_187_	23	0.34 ± 0.08	0.91 ± 0.01	0.28 ± 0.14
	30	0.27 ± 0.01	0.92 ± 0.01	0.20 ± 0.00
	37	0.27 ± 0.01	0.92 ± 0.00	0.19 ± 0.01
InG_253_	23	1.05 ± 0.15	0.81 ± 0.02	0.41 ± 0.02
	30	1.27 ± 0.09	0.79 ± 0.01	0.47 ± 0.01
	37	1.05 ± 0.09	0.81 ± 0.01	0.41 ± 0.02
HiG_427_	23	172.01 ± 56.48	0.23 ± 0.08	12.98 ± 0.40
	30	199.04 ± 12.88	0.19 ± 0.01	11.31 ± 0.13
	37	160.09 ± 54.45	0.25 ± 0.08	11.89 ± 0.54
LoG_495_	23	286.76 ± 16.62	0.20 ± 0.01	76.35 ± 2.61
	30	296.31 ± 53.91	0.20 ± 0.05	67.61 ± 2.65
	37	280.72 ± 40.34	0.20 ± 0.04	61.75 ± 3.48

**Table 3:** Ostwald–de Waele parameters and zero-shear viscosity for all sodium alginates in aqueous solution at different concentrations and 23 °C.

Sodium Alginate	Alginate % (*w*/*w*)	*K* (Pas)	*n*	*η*_0_ (Pas)
LoG_187_	4	0.34 ± 0.08	0.91 ± 0.01	0.28 ± 0.14
	6	1.04 ± 0.06	0.86 ± 0.01	0.67 ± 0.07
	8	3.52 ± 0.49	0.74 ± 0.03	1.42 ± 0.09
InG_253_	4	1.05 ± 0.15	0.81 ± 0.02	0.41 ± 0.02
	6	12.12 ± 4.12	0.59 ± 0.07	1.83 ± 0.08
	8	39.00 ± 3.06	0.46 ± 0.01	5.02 ± 0.43
HiG_427_	4	172.01 ± 56.48	0.23 ± 0.08	12.98 ± 0.40
	6	324.01 ± 61.41	0.25 ± 0.04	58.88 ± 2.53
	7	524.15 ± 39.15	0.18 ± 0.02	93.02 ± 2.87
LoG_495_	2	10.03 ± 0.03	0.54 ± 0.00	2.65 ± 0.20
	4	286.76 ± 16.62	0.20 ± 0.01	76.35 ± 2.61
	5	341.65 ± 73.41	0.24 ± 0.06	146.39 ± 11.98

**Table 4:** Printability parameters for uncross-linked (not shady) and cross-linked (shady) alginate imprint structures (*n* = 3 per group).

Sodium Alginate	Alginate % (*w*/*w*)	*D_s_* (mm)	*D_exp_* (mm)	p (mm)	β (mm^2^)	P_f_	P_p_	$\mathrm{Shrinkage}\;\bar{S}$ (%)
LoG_187_	4	Not printable						
InG_253_	4							
HiG_427_	4	0.43 ± 0.02	1.06 ± 0.05	13.93 ± 0.31	14.44 ± 0.55	2.47 ± 0.11	0.84 ± 0.01	23.86
			0.57 ± 0.04	11.38 ± 0.55	9.36 ± 0.92	1.32 ± 0.09	0.87 ± 0.00	
	6	0.43 ± 0.02	1.01 ± 0.07	14.08 ± 0.30	14.26 ± 0.71	2.36 ± 0.17	0.87 ± 0.01	17.44
			0.62 ± 0.02	12.00 ± 0.11	10.75 ± 0.27	1.46 ± 0.05	0.84 ± 0.01	
LoG_495_	4	0.42 ± 0.01	1.13 ± 0.06	13.84 ± 0.15	14.10 ± 0.26	2.71 ± 0.13	0.85 ± 0.04	36.06
			0.45 ± 0.02	9.86 ± 0.13	6.97 ± 0.19	1.08 ± 0.06	0.87 ± 0.01	
Intended dimension parameters: perimeter = 20 mm; area = 25 mm^2^								

## Data Availability

Not applicable.
